# Angiogenic Effects of Human Dental Pulp and Bone Marrow-Derived Mesenchymal Stromal Cells and their Extracellular Vesicles

**DOI:** 10.3390/cells9020312

**Published:** 2020-01-28

**Authors:** Greet Merckx, Baharak Hosseinkhani, Sören Kuypers, Sarah Deville, Joy Irobi, Inge Nelissen, Luc Michiels, Ivo Lambrichts, Annelies Bronckaers

**Affiliations:** 1 UHasselt - Hasselt University, Faculty of Medicine and Life Sciences, Biomedical Research Institute (BIOMED), Agoralaan, 3590 Diepenbeek, Belgium; 2 Flemish Institute for Technological Research (VITO), Health Department, Boeretang, 2400 Mol, Belgium

**Keywords:** extracellular vesicles, bone marrow-derived mesenchymal stromal cells, dental pulp stromal cells, angiogenesis

## Abstract

Blood vessel formation or angiogenesis is a key process for successful tooth regeneration. Bone marrow-derived mesenchymal stromal cells (BM-MSCs) possess paracrine proangiogenic properties, which are, at least partially, induced by their extracellular vesicles (EVs). However, the isolation of BM-MSCs is associated with several drawbacks, which could be overcome by MSC-like cells of the teeth, called dental pulp stromal cells (DPSCs). This study aims to compare the angiogenic content and functions of DPSC and BM-MSC EVs and conditioned medium (CM). The angiogenic protein profile of DPSC- and BM-MSC-derived EVs, CM and EV-depleted CM was screened by an antibody array and confirmed by ELISA. Functional angiogenic effects were tested in transwell migration and chicken chorioallantoic membrane assays. All secretion fractions contained several pro- and anti-angiogenic proteins and induced in vitro endothelial cell motility. This chemotactic potential was higher for (EV-depleted) CM, compared to EVs with a stronger effect for BM-MSCs. Finally, BM-MSC CM, but not DPSC CM, nor EVs, increased in ovo angiogenesis. In conclusion, we showed that DPSCs are less potent in relation to endothelial cell chemotaxis and in ovo neovascularization, compared to BM-MSCs, which emphasizes the importance of choice of cell type and secretion fraction for stem cell-based regenerative therapies in inducing angiogenesis.

## 1. Introduction

Despite the strong progress in professional health care, current therapies are not able to regenerate the original physiological structure and function of damaged teeth. Therefore, tooth loss remains a major public health issue with a huge economic and social burden [1]. Especially regeneration of tooth pulp is intensively studied, since the current endodontic procedures consist of replacing infected pulp with inorganic materials (such as Gutta-percha), which results in a devitalized (dead) tooth [2]. Pulp removal structurally weakens the tooth and causes a higher susceptibility to infections and fractures [3]. The most important issue that must be considered for pulp tissue regeneration is angiogenesis, as an insufficient blood supply leads to necrosis [4,5]. Angiogenesis is the formation of new blood vessels from pre-existing ones by the sequence of extracellular matrix (ECM) degradation, endothelial cell proliferation, migration, tube formation and vessel maturation with the attraction and attachment of mural cells. This process is tightly regulated by the balance between pro- and anti-angiogenic factors, such as vascular endothelial growth factor (VEGF) and matrix metalloproteinases (MMPs) on one side, and endostatin and tissue inhibitor of metalloproteinases (TIMPs) on the other side [6]. Several studies have been focussing on the administration of proangiogenic mediators to patients suffering from diseases associated with inadequate vascularization, such as stroke and myocardial infarction. However, their success rates were disappointing, probably caused by the limited half-life of recombinant proteins [7].

Alternatively, the application of stem cell therapy in patients could offer a continuous source of angiogenic factors. Paracrine proangiogenic properties have already been assigned to mesenchymal stromal cells (MSCs), both in vitro and in vivo [8,9]. While most studies have focused on bone marrow-derived MSCs (BM-MSCs), several drawbacks are associated with these stem cells, including their painful and invasive isolation [10]. These limitations could be overcome using dental pulp stromal cells (DPSCs), neural crest-derived cells present in the dental pulp cavity. They are considered MSC-like cells based on their plastic adherence, surface marker expression and trilineage differentiation potential into osteoblasts, chondroblasts and adipoblasts [11]. Compared to BM-MSCs, they can easily be isolated from deciduous and permanent teeth, and have superior proliferation and differentiation capacities [12,13]. In addition, DPSCs express a plethora of proangiogenic factors and induce angiogenesis, at least partially in a paracrine way, since both cells and their conditioned medium (CM) can promote in vitro endothelial cell migration, tube formation, in vivo angiogenesis and tissue regeneration [14,15].

Stem-cell based therapies have been shown to obtain promising results in (pre-) clinical studies [16,17]. However, stem cell transplantation might be associated with complications, including immune rejection and tumour formation [18]. More recent studies have suggested that extracellular vesicles (EVs) derived from BM-MSCs might possess proangiogenic paracrine effects [19,20,21]. These small membrane-bound vesicles comprise exosomes, microvesicles and apoptotic bodies, which can be differentiated based on size and origin. They contain proteins, DNA and miRNA, and play an important role in cell-cell communication. Since they can be stored stably for several months as ready-to-use, off-the-shelf products and cell-associated complications can be avoided, their potential therapeutic application in regenerative medicine is currently extensively investigated [22,23]. BM-MSC EVs induce in vitro and in vivo angiogenesis, and improve functional recovery in animal models of myocardial infarction and skeletal muscle injury [19,20,24]. Studies on the angiogenic capacity of DPSC EVs are scarce. Xian et al. showed a positive effect of DPSC-derived exosomes on endothelial cell proliferation and tube formation [25]. These results could be confirmed by Gonzalez-King et al. in an in vitro tube formation assay and in vivo mouse matrigel plug model [26]. 

The goal of our research was to evaluate whether DPSCs and their associated EVs could act better than BM-MSCs in the induction of angiogenesis for eventual tissue regeneration. This study specifically focussed on endothelial cell migration, as one of the key steps in the angiogenic process, and in ovo stimulation of new blood vessel formation. EVs from both cell types were thoroughly characterized by nanoparticle tracking analysis (NTA), western blot, high-resolution flow cytometry (HR-FCM) and transmission electron microscopy (TEM). Their angiogenic content was identified by a protein array and confirmed by ELISA. Moreover, time-dependent uptake of DPSC- and BM-MSC-derived EVs by endothelial cells was measured by fluorescence microscopy. Finally, the functional effects of EVs on in vitro endothelial cell migration and in ovo angiogenesis were compared with those of soluble proteins secreted by DPSCs and BM-MSCs. To our knowledge, this is the first study that compares the angiogenic effects between CM containing soluble factors and EVs, CM depleted of EVs and isolated EVs of DPSCs and BM-MSCs. 

## 2. Materials and Methods

### 2.1. Cell Isolation and Culture

Dental pulp tissue was obtained from wisdom teeth of thirteen healthy donors (14–23 years of age, both sexes) undergoing tooth extraction for orthodontic or therapeutic reasons at Ziekenhuis Oost-Limburg (ZOL, Genk, Belgium). The study was conducted in accordance with the Declaration of Helsinki, and the study protocol was approved by the medical ethical committee of Hasselt University (Belgium, protocol 13/0104U, date of approval 3 February 2014). Written informed consent was given by all participants or guardians in case of minors. DPSCs were isolated by the explant method, as previously described [27]. Cells were cultured in Minimal Essential Medium, alpha modification (α-MEM, Sigma-Aldrich, St-Louis, MO, USA) enriched with 10% heat-inactivated foetal bovine serum (FBS, Biochrom AG, Berlin, Germany), 2 mM l-glutamine, 100 U/mL penicillin and 100 µg/mL streptomycin (Sigma-Aldrich). Cells between passages three to seven were used.

BM-MSCs of three different donors (both males and females), between 6 and 12 years old, were kindly provided by Prof. Dr. Cathérine Verfaillie (Stem Cell Institute, KU Leuven, Leuven, Belgium). Cells were maintained in high-glucose Dulbecco’s Modified Eagle’s Medium (DMEM, Sigma-Aldrich) supplemented with 10% heat-inactivated FBS, 100 U/mL penicillin and 100 µg/mL streptomycin. The experiments were performed on passages six to ten. 

Human umbilical vein endothelial cells (HUVECs) were purchased from Corning (HUVEC-2, #354151, Lasne, Belgium) and cultured in endothelial cell growth medium (EBM-2, Lonza, Walkersville, MD, USA) enriched with growth factors (EGM-2 SingleQuots^TM^, Lonza) and 10% heat-inactivated FBS until passage ten.

The cells were screened monthly for mycoplasma infection with the PlasmoTest^TM^ kit (InvivoGen, Toulouse, France). Research was only performed on mycoplasma negative samples. 

### 2.2. Isolation of Extracellular Vesicles by Differential Ultracentrifugation

DPSCs and BM-MSCs were seeded at 20,000 cells/cm^2^ in T75 polystyrene culture flasks (Corning) with 15 mL of their standard culture medium for overnight adherence. Afterwards, cells were washed twice in 5 mL PBS, and 15 mL serum-free low-glucose DMEM medium (Sigma-Aldrich), supplemented with 1 mM sodium pyruvate (Sigma-Aldrich), 2 mM l-glutamine, 100 U/mL penicillin and 100 µg/mL streptomycin was added. After 48 h, CM was collected and EVs were freshly isolated by differential ultracentrifugation at 4 °C. Cell debris was removed by centrifugation at 269× *g* for 6 min. All cell-derived EV populations (exosomes, microvesicles and apoptotic bodies) were pelleted in polycarbonate tubes (#355618, Beckman Coulter, Brea, CA, USA) by ultracentrifugation at 100,000× *g* and braking 2 during 3 h using an L-90 Beckman centrifuge with a Ti-70 rotor (Beckman Instruments, Fullerton, CA, USA, k-factor: 220.1). The resulting supernatant was used as EV-depleted CM. The EV-enriched fraction derived from 25 mL CM was resuspended in 869 µL DMEM medium, 200 µL PBS or 250 µL RIPA buffer (50 mM Tris (pH 8.0), 150 mM NaCl, 0.5% sodium deoxycholate, 0.1% sodium dodecyl sulphate (SDS), 1% Triton X-100) supplemented with Protease Inhibitor Cocktail (#05 892 970 001, Roche, Basel, Switzerland). All sample fractions, except for lysed EVs, were filtered (0.2 µm, #83.1826.001, Sarstedt, Nümbrecht, Germany) for sterility and stored at −80 °C for downstream applications. The number of living cells at time of CM collection was determined via the trypan blue exclusion method and no difference between both stem cells could be detected with a cell viability of more than 95% (Figure S1). To allow proper comparison between the protein content and functional effects of EV-depleted CM, CM and EVs, concentration of CM and EV-depleted CM was needed. This was done in Vivaspin centrifugation filters (3000 Da, Sartorius, Brussels, Belgium) at 5000× *g* and 4 °C. In this way, 1 mL of 25X CM was obtained, which corresponded to 1 mL of 1X EVs, since both fractions were produced by the same amount of cells. 

### 2.3. Western Blotting

Protein concentrations of DPSC and BM-MSC EVs resuspended in RIPA buffer were measured by Pierce BCA Protein Assay Reagent Kit (Thermo Fisher Scientific, Erembodegem, Belgium) conform the manufacturer’s instructions. Samples containing 2.6 µg protein were diluted in 5X SDS loading buffer (10% SDS, 50% glycerol, 0.325 M Tris-HCl (pH 6.8) and 0.025% bromophenol blue), loaded on 12% polyacrylamide gels and transferred to polyvinylidene fluoride (PVDF) membranes. After blocking with 5% non-fat dry milk (Marvel, Thame, UK) in PBS for 2 h at room temperature using gentle shaking, the blots were incubated overnight at 4 °C with primary antibodies against CD9 (Ts9, #10626D), CD63 (Ts63, #10628D), CD81 (M38, #10630D) (all 1/1000, Thermo Fisher Scientific), Annexin II (1/500, C-10, #sc-28385, Santa Cruz, Heidelberg, Germany) and Bax (1/1000, E63, #ab32503, Abcam, Cambridge, UK). Rabbit anti-mouse (1/2000, #P0260) or goat anti-rabbit (1/1000, #P0448) horseradish peroxidase-conjugated secondary antibody (both Agilent, Heverlee, Belgium) was added for 1 h at room temperature using gentle shaking. All antibodies were diluted in blocking buffer and washing steps were performed in 0.1% Tween 20 in PBS. The bands were visualized by WesternBright Sirius HRP substrate (Advansta, CA, USA) and images were taken with the ImageQuant LAS 4000 Mini (GE Healthcare, Diegem, Belgium). Equal protein amounts of cell lysates from DPSCs and BM-MSCs served as positive controls. All experiments were performed under non-reducing conditions, except for Annexin II and Bax. 

### 2.4. Nanoparticle Tracking Analysis (NTA)

Particle size and concentration of DPSC and BM-MSC EVs were measured by a NanoSight NS300 device equipped with a 532 nm laser (Malvern Panalytical, Worcester, UK) based on the light scattering of particles in suspension undergoing Brownian movement. EV suspensions were diluted with PBS over a range of concentrations to obtain between 10 and 100 particles per frame. Each sample was measured five times for 60 s at 25 °C with manual shutter at camera level 16. Data were analysed by NTA software 3.2 (Malvern) with manual gain adjustments and detection threshold 6–21. 

### 2.5. Transmission Electron Microscopy (TEM) 

Five μL of EV sample solution in 2% glutaraldehyde was placed on Formvar–copper coated EM grids (Polyscience, Inc, Warrington, PA, USA) for 15 min. Afterwards, the samples were washed twice with distilled H_2_O and grids were negatively contrasted with 2% uranyl acetate (Sigma-Aldrich). The images from each grid were captured using a Tecnai G2 TEM (Tecnai G2 spirit twin, FEI, Eindhoven, the Netherlands) at 120 kV and analysed with FEI imaging software (TEM Imaging and Analysis version 3.2 SP4 build 419).

### 2.6. High-Resolution Flow Cytometry (HR-FCM)

Prior to HR-FCM analysis, DPSC and BM-MSC EV isolates were fluorescently stained with carboxyfluorescein diacetate, succinimidyl ester (CFDA-SE, #V12883, Thermo Fisher Scientific) as previously described [28,29]. Briefly, 30 µL of EVs were incubated with 3 µL of CFDA-SE to a final concentration of 10 µM for 1 h at room temperature in the dark. Thereafter, 0.5 µg of fluorescently labelled antibodies were added and samples were incubated for 1 h at room temperature in the dark for the detection of CD34 (CD34-PeCy5, #555823, BD, Erembodegem, Belgium), CD45 (CD45-PeCy5, #555484, BD), CD44 (CD44-PeCy5, #103009, BioLegend, San Diego, USA), CD73 (CD73-BrilliantBlue700, #74600, BD) and CD90 (CD90-PeCy5, #328111, BioLegend). The stained EVs were further purified overnight by bottom-up density gradient centrifugation using an iodixanol gradient. For this, samples were diluted to a final volume of 300 µL by adding 0.1 µm filtered PBS and transferred to 5 mL open-top thinwall polyallomer tubes (#103242, Beckman Coulter). Thereafter, samples were mixed with 1 mL of 60% iodixanol (#07820, Optiprep, StemCELL Technologies, Cologne, Germany). This mixture was overlaid with 700 µL of 40% iodixanol, 700 µL of 30% iodixanol and 2 mL of 10% iodixanol. Iodixanol dilutions were prepared by dilution of the 60% iodixanol to 40% iodixanol using a homogenization buffer containing 6 mM EDTA, 60 mM Tris-HCl and 0.25 mM sucrose (pH 7.4) and subsequent dilutions were performed by dilution of 40% iodixanol to 30% iodixanol and 10% iodixanol using a homogenization buffer containing 1 mM EDTA, 10 mM Tris-HCl and 0.25 mM sucrose (pH 7.4). Thereafter, the samples were centrifuged for 14 h at 367,600× *g* and 4 °C using an Optima XPN-80 ultracentrifuge and SW55 Ti rotor (Beckman Coulter, k-factor: 48.5) and moderate braking (5/5). 

Fractions of 480 µL were collected and measured on a BD Influx flow cytometer equipped with a high power 488 nm laser (200 mW), and a small-particle detector for high sensitivity forward scatter detection was used for analysis. The device utilizes a highly sensitive fluorescence trigger to measure the EVs. The threshold of the FL-1 fluorescent channel (BP530/40) was set to 0.30, based on the measurement of 0.1 µm filtered PBS, to exclude background events. Yellow-Green fluorescent beads of 100 and 200 nm (#F8803 and F8811, Thermo Fisher Scientific) were used for setting gates to allow for small particle detection. The density of the iodixanol fractions was determined by absorbance spectroscopy at 340 nm (Clariostar, BMG Labtech, Ortenberg, Germany). Multiple 1:1 aqueous dilutions of iodixanol solutions (5–40%) were prepared and the absorbance was measured. Using this standard curve, the density of the fractions collected from a control iodixanol gradient was determined. Fractions corresponding to 1.10 g/mL were used for further analysis (Figure S2). The samples were diluted 1/20 in PBS, measured at a flow rate of approximately 10 µL/min and 50,000 events were acquired to determine the abundance of the selected surface markers. Three technical replicates were performed for every EV measurement. 

### 2.7. Cell Surface Area of DPSCs and BM-MSCs

The cells were seeded on coverslips in standard culture medium at a density of 2,632 cells/cm^2^ and fixed with 4% paraformaldehyde (PFA) after three days. Cell membranes and nuclei were stained with Alexa Fluor 647-labelled Wheat Germ Agglutinin (WGA, Thermo Fisher Scientific) and 4′,6-diamidino-2-phenylindole (DAPI, Thermo Fisher Scientific) during 10 min at room temperature in the dark, respectively. Coverslips were mounted by Fluoromount-G^TM^ (Thermo Fisher Scientific). Fifteen pictures were taken per sample with a Leica DM4000 B Microscope (Leica Microsystems, Wetzlar, Germany) at a final magnification of 200× and the mean cell surface areas were calculated with ImageJ Software. 

### 2.8. Angiogenesis Antibody Array

The angiogenic content of lysed DPSC and BM-MSC EVs and their corresponding EV-depleted CM was screened with the Proteome Profiler^TM^ Human Angiogenesis Array Kit (R&D Systems, Minneapolis, MN, USA), following the manufacturer’s protocol. The membranes were incubated with 1 mL of lysed EVs and EV-depleted CM concentrated from 25 mL CM of three different donors per cell type. Signal density was visualized and quantified by ImageQuant LAS 4000 Mini equipped with ImageQuant TL software. For every protein, signals of the negative control spots were subtracted and the corrected signal densities were normalized to the positive reference spots. The resulting relative pixel densities were represented in a heat map. 

### 2.9. Enzyme-Linked Immunosorbent Assay (ELISA)

To confirm the semi-quantitative results of the angiogenesis array, protein concentrations of selected pro- and anti-angiogenic factors were measured in the CM, EV-depleted CM and (lysed) EVs of DPSCs and BM-MSCs by ELISA. ELISA kits for VEGF (#DY293B), insulin-like growth factor-binding protein-1 (IGFBP-1, #DY871), TIMP-1 (#DY970), angiopoietin-1 (Angpt-1, #DY923) (R&D Systems) and IGFBP-3 (#ab217607, Abcam) were used conform the manufacturer’s guidelines. Data were normalized to the number of seeded cells.

### 2.10. Transwell Migration Assay

The chemotactic capacity of EVs, CM and EV-depleted CM of DPSCs and BM-MSCs on endothelial cells was tested in a Boyden chamber migration assay. HUVECs were seeded at a density of 3,500 cells/mm^2^ in serum-free low-glucose DMEM medium in the inserts of a HTS Transwell-96 permeable support with pores of 8 µm (Corning). Different concentrations of CM, EV-depleted CM (25X, 5X, 1X, 1/5, 1/25) and EVs (1X, 1/2, 1/4, 1/8) in DMEM medium of both stem cell types were added to the lower compartment in a volume of 100 µL. Low-glucose DMEM medium with or without 10% FBS in the lower wells was used as positive and negative control, respectively. After 24 h of incubation, transmigrated cells were fixed by 4% PFA and stained with 0.1% crystal violet (Sigma-Aldrich) in 70% ethanol. Representative pictures were taken by a Nikon eclipse TS100 inverted microscope with a Jenoptik ProgRes C3 camera (Jenoptik, Jena, Germany). Migration was quantified as the mean area percentage covered on the lower side of the membrane by Axiovision 4.6 Software (Carl Zeiss Vision, Aalen, Germany). 

### 2.11. Internalization of EVs by Endothelial Cells

To test the cellular uptake of EVs by HUVECs, DPSC and BM-MSC EVs derived from 25 mL CM were labelled with 10 µL of 0.1 µg/µL DiI (Sigma-Aldrich) for 10 min at 37 °C in 1 mL DMEM medium in the dark. Excess dye was removed by ultracentrifugation at 100,000× *g* during 3 h at 4 °C in 25 mL serum-free low-glucose DMEM medium using an L-90 Beckman centrifuge with a Ti-70 rotor and braking 2. DiI staining of the isolated particles was confirmed by NTA with a 532 nm laser. For each sample, five measurements of 60 s at 25 °C with camera level 16, syringe pump speed 80 and detection threshold 4–5 were performed. After overnight adherence of 5,000 HUVECs/cm^2^ on coverslips in a 48-well plate, cells were incubated with 150 µL DiI labelled EVs for 1, 3, 6 or 24 h. DiI labelled serum-free DMEM medium without EVs, treated in the same way as the EV fraction, was used as negative control sample. After fixation with 4% PFA, the cell membranes were stained with Alexa Fluor 647-labelled WGA and nuclei were counterstained with DAPI for 10 min, at room temperature, in the dark. Coverslips were mounted with Fluorescent mounting medium (Dako, Glostryp, Denmark). At least 7 pictures were taken per sample with a Leica DM4000 B Microscope at a final magnification of 200×. The mean integrated fluorescent density signal per cell was quantified with ImageJ software. Additional pictures were taken by a Zeiss LSM 880 Confocal Microscope. 

### 2.12. Chicken Chorioallantoic Membrane (CAM) Assay 

The angiogenic capacity of DPSCs and BM-MSCs was compared in an in ovo CAM assay as previously described [30]. At day 9 of the embryonic development (E9), CAMs were incubated with plastic discs containing 30 µL of EVs or 25X concentrated CM derived from DPSCs and BM-MSCs. All samples were diluted twice in growth factor-reduced Matrigel (Corning) and allowed to solidify for 1–2 h at 37 °C before being placed on the CAM. Recombinant human insulin-like growth factor-2 (IGF-2) in 25X concentrated DMEM medium (500 ng, ImmunoTools, Friesoythe, Germany), and standard or 25X concentrated DMEM medium in 50% Matrigel were used as positive, and negative controls, respectively.

### 2.13. Statistical Analysis

ELISA and transwell migration results were analysed with SAS JMP Pro 13.2.0 Software (SAS institute, Cary, NC, USA). The data were log-transformed to reduce differences in variability between experimental groups when needed and normality was confirmed based on the Normal Quantile plot. A mixed model ANOVA was combined with a Tukey–Kramer multiple comparison post-hoc test with experimental conditions as fixed effect. To correct for variation between experiments and interdependence of CM, EV-depleted CM and EVs derived from the same stem cell donor, experiment or donor was added as random effect in the model. Other data were statistically analysed with GraphPad Prism 8 Software (Graphpad Software, La Jolla, CA, USA). After testing data normality using the D’Agostino and Pearson omnibus normality test, both stem cell types were compared using the Mann-Whitney U test or two-way ANOVA with Bonferroni multiple comparison post-hoc test. To compare multiple experimental groups, a Kruskal-Wallis test with Dunn’s post-hoc test or one-way ANOVA with Sidak’s post-hoc test was performed. P-values below 0.05 were considered statistically significant. All quantitative results were expressed as mean ± standard error of the mean (S.E.M.).

### 2.14. EV Track

We have submitted all relevant data of our experiments to the EV-TRACK knowledgebase (EV-TRACK ID: EV190084) (Van Deun, J.; et al. EV-TRACK: transparent reporting and centralizing knowledge in extracellular vesicle research. *Nat. Methods* **2017**, *14*, 228–232). The EV-METRIC score is 78%.

## 3. Results

### 3.1. Differential EV Secretion Pattern of DPSCs and BM-MSCs

**Figure 1:**
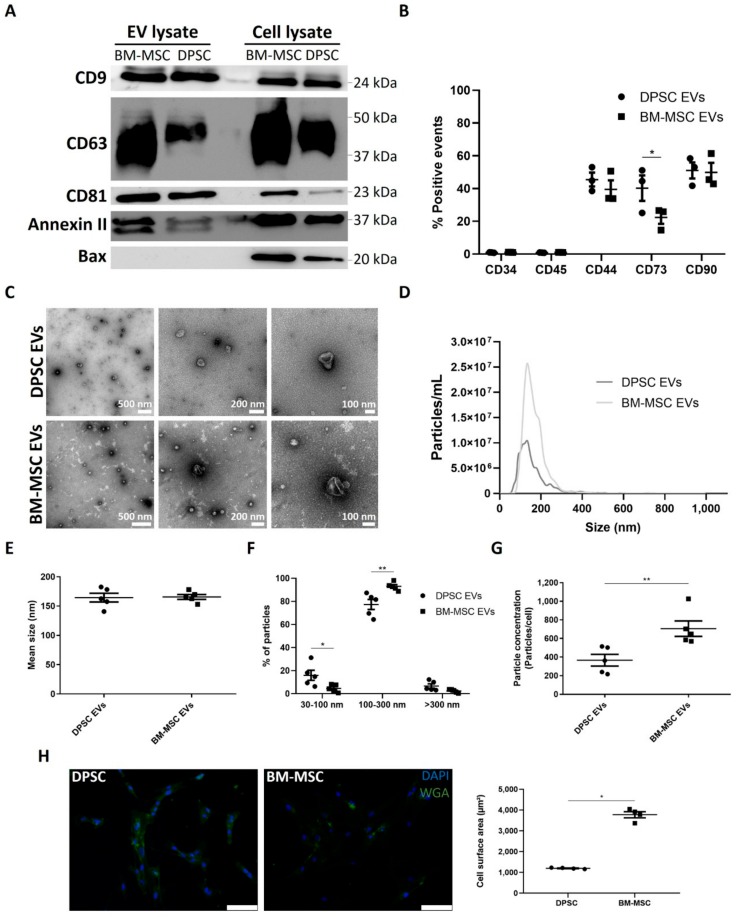
Characterization of extracellular vesicles (EVs) from dental pulp stromal cells (DPSCs) and bone marrow-derived mesenchymal stromal cells (BM-MSCs) isolated by differential ultracentrifugation. The purity of DPSC and BM-MSC EV samples was tested using western blot for classical EV markers CD9, CD63, CD81 and Annexin II and non-EV marker Bax with cell lysates as positive control samples (**A**). High-resolution flow cytometry for mesenchymal surface markers CD44, CD73 and CD90, and endothelial and hematopoietic surface antigens CD34 and CD45 confirmed their mesenchymal source of origin (**B**, *n* = 3, 3 different donors). EV morphology was ultrastructurally analysed by transmission electron microscopy (**C**). Nanoparticle tracking analysis revealed the average size distribution profile for both EV types (**D**, *n* = 5, 5 different donors (DPSCs) or five different isolations from three different donors (BM-MSCs)). While their mean particle size was similar (**E**), the fractions of small and intermediate particles significantly differed between both stem cell types (**F**) and BM-MSCs secreted more particles per cell (**G**). In addition, the active cell surface area was higher for BM-MSCs compared to DPSCs. Wheat germ agglutinin (WGA) and DAPI were used to stain stem cell membranes and nuclei, respectively (**H**, *n* = 4, 3 (BM-MSCs) or 4 (DPSCs) different donors, scale bar = 100 µm). Data are presented as mean ± S.E.M. * *p* < 0.05, ** *p* < 0.01 as determined by two-way ANOVA combined with Bonferonni post-hoc test (**B**, **F**) or Mann-Whitney U test (**G**–**H**).

The EV pellet consisting of small to large vesicles, isolated from DPSCs and BM-MSCs by differential ultracentrifugation, was characterized following the International Society for Extracellular Vesicles (ISEV) guidelines [31,32] (Figure 1). The expression of classical EV surface markers CD9, CD63, CD81 and cytoplasmic factor Annexin II was demonstrated by western blot. No expression of non-EV mitochondrial protein Bax could be detected in EVs that were derived from both cell types, while their cellular counterparts were positive (Figure 1A). Flow cytometry confirmed the mesenchymal origin of the EVs by the presence of mesenchymal antigens CD44, CD73 and CD90 and the absence of endothelial and hematopoietic markers CD34 and CD45. Significantly less CD73-positive EVs were secreted by BM-MSCs compared to DPSCs (Figure 1B and Figure S3). At cellular level, DPSCs and BM-MSCs were positive for CD44, CD73 and CD90 (≥95%) and negative for CD34 and CD45 (≤5%) (Representative plots in Figure S3). 

TEM and NTA revealed a heterogeneous EV population with a typical cup-shaped morphology (Figure 1C) and particle size, between 50 and 300 nm (Figure 1D). While, the mean size of both secreted DPSC and BM-MSC EVs was similar (164.4 ± 7.5 vs. 165.6 ± 4.1 nm, Figure 1E), the fraction of small particles (30–100 nm) was larger for DPSCs (15.96 ± 4.33 vs. 4.64 ± 1.46%). In contrast, BM-MSCs secreted more intermediate particles (100–300 nm, 77.32 ± 4.39 vs. 93.00 ± 1.53%). The number of large particles (>300 nm) was minimal for both stem cells (<6%) (Figure 1F). Detection of particles larger than 200 nm in the samples suggests the aggregation of multiple smaller particles after the 0.2 µm filtration step. Furthermore, BM-MSCs secreted about two times more EVs compared to DPSCs (706 ± 83 vs. 367 ± 63 particles per cell, Figure 1G) and had a three-fold larger active cell surface area (3777 ± 148 vs. 1198 ± 16 µm^2^, Figure 1H). Collectively, these data confirm the successful isolation of EVs from both stem cell types with a higher particle secretion for BM-MSCs. 

### 3.2. DPSC- and BM-MSC-Derived EVs Contain Pro- and Anti-Angiogenic Factors

**Figure 2:**
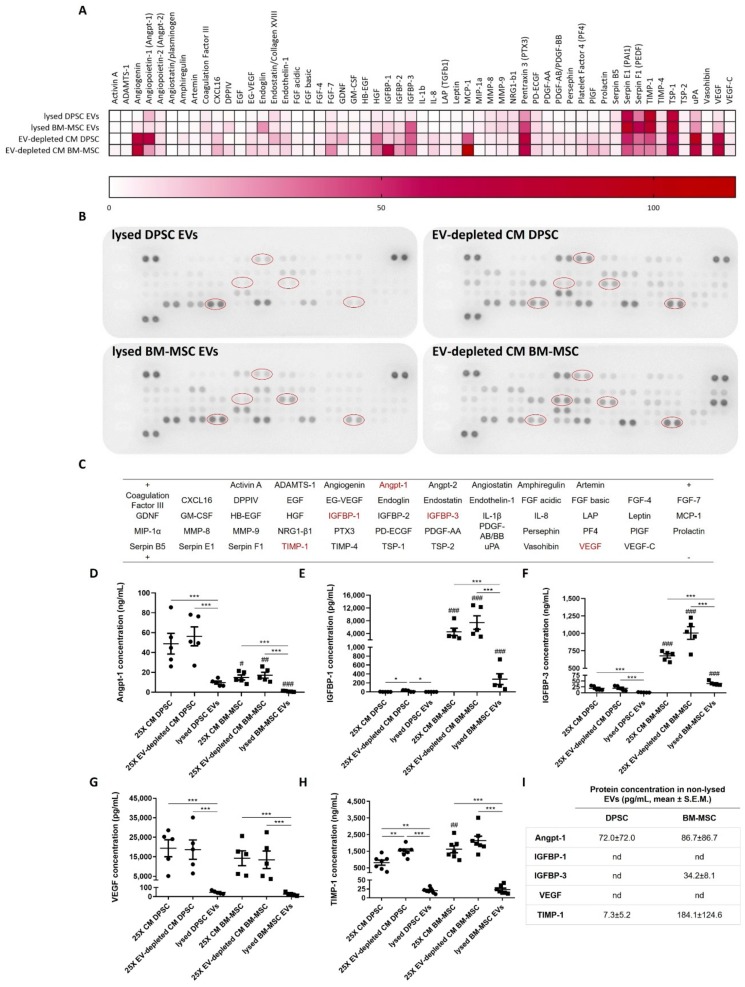
Angiogenic protein profiles of CM, EV-depleted CM, and lysed and non-lysed EVs of DPSCs and BM-MSCs. A screening for 55 pro- and anti-angiogenic factors was performed on EV-depleted CM and lysed EVs derived from DPSCs and BM-MSCs (*n* = 3, 3 different donors). Relative pixel density was expressed as percentage after subtraction of the negative control and normalization to positive reference spots (indicated as ‘−’ and ‘+’) and displayed in a heat map (**A**). Representative antibody blots are presented for each condition (**B**), with the corresponding protein locations (**C**). Semi-quantitative results of proteins angiopoietin-1 (Angpt-1) (**D**), IGFBP-1 (**E**), IGFBP-3 (**F**), VEGF (**G**) (*n* = 5, 5 different donors (DPSCs) or 5 different isolations of 3 different donors (BM-MSCs)) and TIMP-1 (**H**) (*n* = 7, 7 different donors (DPSCs) or 7 different isolations of 3 different donors (BM-MSCs)) were confirmed with ELISA on CM, EV-depleted CM and (lysed) EVs of both stem cell types (factors indicated in red). Limited or no expression of these angiogenic factors could be detected in non-lysed EV samples, suggesting that the EV-associated factors are located intravesicularly (**I**, nd = not detectable). Significant differences in angiogenic protein content between stem cell types and secretion fractions could be observed as determined with mixed model ANOVA. Quantitative data are shown as mean ± S.E.M. * *p* < 0.05, ** *p* < 0.01, *** *p* < 0.0001 between EVs, CM and EV-depleted CM, ^#^ *p* < 0.05, ^##^ *p* < 0.01, ^###^ *p* < 0.0001 between DPSCs and BM-MSCs.

In order to study the angiogenic profile of EVs, derived from DPSCs and BM-MSCs, their content of pro- and anti-angiogenic factors was elucidated (Figure 2). As an initial step, an antibody array for 55 proteins was performed on lysed EVs and on corresponding EV-depleted CM of both stem cell types. A wide range of pro- and anti-angiogenic factors could be detected in EVs and EV-depleted CM as showed in a heat map (Figure 2A–C). Most angiogenic molecules, such as angiogenin, angiopoietin-1 (Angpt-1), hepatocyte growth factor (HGF), insulin-like growth factor-binding proteins (IGFBPs), monocyte chemoattractant protein-1 (MCP-1), urokinase plasminogen activator (uPA) and VEGF, were mainly expressed in EV-depleted CM. In contrast, pro- and anti-angiogenic mediators pentraxin 3, serpin E1 and F1, TIMP-1 and thrombospondin-1 (TSP-1) were abundantly present in both EV-depleted CM and lysed EVs. For some proteins, variation in expression could be observed between DPSCs and BM-MSCs, including Angpt-1, IGFBP-1, IGFBP-3 and MCP-1. To confirm these semi-quantitative results for selected differentially expressed proteins, ELISAs for the pro- and anti-angiogenic molecules Angpt-1, IGFBP-1, IGFBP-3, TIMP-1 and VEGF were performed on CM, EV-depleted CM and EVs of DPSCs and BM-MSCs. In addition, to identify whether an angiogenic protein was present inside the vesicles or on their membrane, both lysed and non-lysed EVs were included in the analysis. The concentrations of all tested factors were higher in CM and EV-depleted CM compared to lysed EVs (Figure 2D–H). BM-MSCs expressed significantly more IGFBP-1 and IGFBP-3 in all fractions compared to DPSCs. For example, 25X CM of BM-MSCs contained 35.3-fold more IGFBP-3 than 25X DPSC CM (677.0 ± 30.8 vs. 19.2 ± 4.4 ng/mL). In lysed BM-MSC EVs, 39.7 ± 3.8 ng/mL IGFBP-3 was detected, while the IGFBP-3 content in DPSC EVs was only 1.6 ± 0.4 ng/mL. In addition, significantly higher TIMP-1 levels could be measured in CM derived from BM-MSCs compared to DPSC CM (1619.0 ± 212.0 vs. 810.6 ± 152.4 ng/mL). Nevertheless, Angpt-1 was 3.3-fold more secreted in DPSC CM compared to BM-MSC CM (48.9 ± 10.4 vs. 14.9 ± 2.5 ng/mL). For lysed EVs, the Angpt-1 concentration was also 10-fold higher for DPSCs in comparison to BM-MSCs (9.6 ± 1.5 vs. 0.8 ± 0.2 ng/mL). There were no differences in VEGF levels between DPSCs and BM-MSCs. The protein concentrations in non-lysed EV samples were limited or undetectable, suggesting that the tested angiogenic factors were located inside the vesicles (Figure 2I). 

### 3.3. Endothelial Cells Are Attracted by DPSCs and BM-MSCs in a Paracrine Way

**Figure 3:**
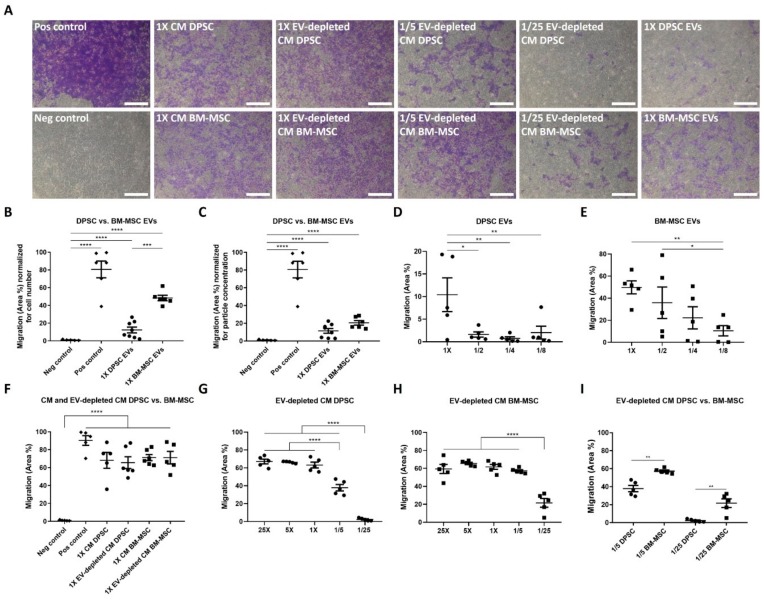
Chemoattractant capacity of DPSC and BM-MSC CM, EV-depleted CM and EVs on endothelial cells. All secretion fractions of DPSCs and BM-MSCs were functionally tested in a transwell migration assay with human umbilical vein endothelial cells (HUVECs). Medium with, or without, 10% serum served as positive, and negative control, respectively. Transmigrated cells were fixed and stained with crystal violet after 24 h (**A**). Both EV types could induce endothelial cell chemotaxis with a stronger capacity for BM-MSCs (**B**), which was eliminated after normalization for their higher particle concentration as measured by NTA (**C**) (*n* = 8, 8 different isolations from 7 different donors (DPSCs), *n* = 6, 6 different isolations from three different donors (BM-MSCs)). This functional effect of DPSC (**D**) and BM-MSC (**E**) EVs followed a dose-dependent course (*n* = 5, 5 different donors (DPSCs) or five different isolations of two different donors (BM-MSCs)). Endothelial cell migration could also be induced by the associated CM and EV-depleted CM (**F**, *n* = 5–6, 5–6 different donors (DPSCs) or 5–6 different isolations of 2–3 different donors (BM-MSCs)) in a concentration-dependent way for EV-depleted CM derived from DPSCs (**G**) and BM-MSCs (**H**). At lower concentrations, the chemotactic effect of BM-MSC EV-depleted CM on endothelial cells was significantly higher compared to DPSCs (**I**) (*n* = 5, 5 different donors (DPSCs) or 5 different isolations of 3 different donors (BM-MSCs)). Scale bars represent 500 µm (**A**) and endothelial cell migration is expressed as the mean area percentage ± S.E.M. (**B**–**I**). * *p* < 0.05, ** *p* < 0.01, *** *p* < 0.001, **** *p* < 0.0001 as determined with mixed model ANOVA.

Since endothelial cell migration is one of the key steps in angiogenesis, the chemotactic paracrine functional effects of DPSC and BM-MSC EVs on endothelial cells were studied in a transwell assay with the different secretion fractions of DPSCs and BM-MSCs in the lower wells (Figure 3A). To make a correct comparison between all fractions, the EV-depleted CM was concentrated 25 times, which corresponds to the volume of the isolated undiluted EVs. EVs from both stem cell types could induce endothelial cell attraction in a dose-dependent way after 24 h (Figure 3B–E). When the migration potential of EVs was calculated per cell number (i.e., seeded BM-MSCs/DPSCs), BM-MSC EVs had a stronger chemotactic effect than DPSC EVs (48.37 ± 3.08 vs. 12.35 ± 3.28%) (Figure 3B). However, in case the migration capacity was normalized to particle concentration, there was no detectable difference observed between DPSCs and BM-MSCs (Figure 3C). For both stem cell types, CM elicited a stronger migration response of endothelial cells compared to EVs. EV depletion of DPSC- and BM-MSC-derived CM had no influence on the chemoattractant capacities of the CM (Figure 3F). In order to quantitatively compare the chemotactic potential of DPSCs and BM-MSCs, dilution series were made. While, EV-depleted CM of both cell types expressed a dose-dependent positive effect on endothelial cell migration (Figure 3G–H), BM-MSCs revealed a stronger potential at lower concentrations, as compared to DPSCs (Figure 3I). For example, 1/25 dilution of EV-depleted CM of BM-MSCs significantly induced migration, while no effect was seen for the same dilution of EV-depleted DPSC CM. Altogether, EVs and soluble factors, secreted by BM-MSCs, had a stronger chemotactic effect on endothelial cells compared to DPSCs.

### 3.4. Time-Dependent Internalization of EVs Derived from DPSCs and BM-MSCs by Endothelial Cells

**Figure 4:**
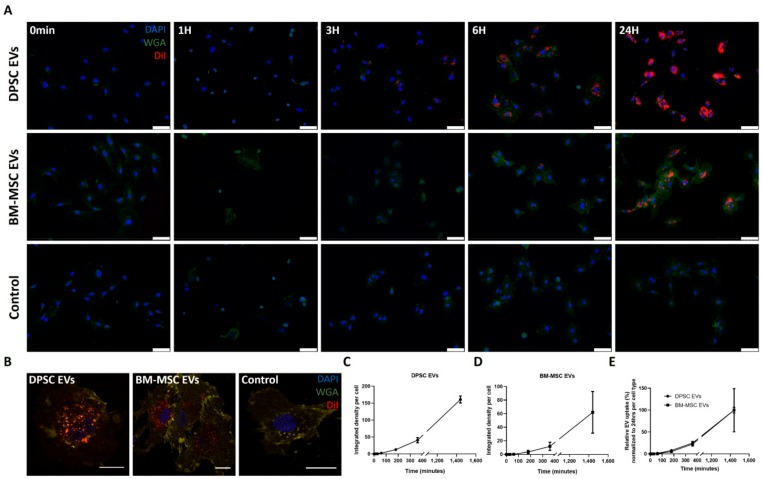
Time-dependent uptake of DPSC- and BM-MSC-derived EVs by endothelial cells. DiI-labelled EVs of DPSCs or BM-MSCs were co-cultured with HUVECs and their uptake was visualized by fluorescence microscopy at different time points (*n* = 3, 3 different donors). EV-free medium labelled with DiI served as control sample (*n* = 3 for 24 h). A clear time-dependent internalization of EVs from both stem cell types could be observed from 3 h onward (**A**) and was confirmed by confocal microscopy after 24 h (**B**). Cell nuclei and membranes of HUVECs were stained with DAPI and wheat germ agglutinin (WGA), respectively. EV uptake was quantified as mean integrated fluorescent density signal per cell for DPSC (**C**) and BM-MSC (**D**) EVs. Both EV types were internalized by HUVECs at similar rates (**E**). Scale bars represent 75 µm (**A**) or 20 µm (**B**) and data are expressed as mean ± S.E.M. (**C**–**E**).

Endothelial cells were incubated with DiI labelled DPSC- and BM-MSC-derived EVs during 24 h (Figure 4), in order to test whether any variation in uptake capacity of both EV types by endothelial cells could explain the differences in chemoattractant effects. Fixation at different time points and analysis using fluorescence microscopy revealed a clear internalization starting from 3 h onward. No uptake of fluorescent label could be detected in the control sample consisting of DiI labelled EV-free medium (Figure 4A). These results were also confirmed by confocal imaging after 24 h (Figure 4B). EV incorporation was quantified as the integrated fluorescent density signal per cell and showed a time-dependent course (Figure 4C,D). To correct for the potential variation in concentration and labelling efficiency between DPSC and BM-MSC EVs, fluorescent density signals were normalized to the final signal measured after 24 h. No differences in EV uptake rates by endothelial cells could be observed between EVs from both stem cells (Figure 4E). 

### 3.5. Paracrine Positive Effects of BM-MSCs, but Not DPSCs, on In Ovo Angiogenesis

**Figure 5:**
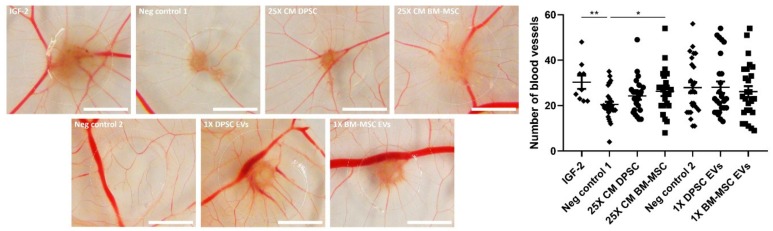
In ovo angiogenic effect of CM and EVs derived from DPSCs and BM-MSCs. Plastic discs containing EVs (*n* = 26 eggs) or 25X concentrated CM of DPSCs (*n* = 24 eggs) and BM-MSCs (*n* = 23 eggs) in Matrigel were placed on the chorioallantoic membrane (CAM) of chicken embryos at embryonic day 9. Concentrated (*n* = 30 eggs) and standard (*n* = 26 eggs) DMEM medium were used as negative controls 1, and 2, respectively, while 500 ng IGF-2 in 25X concentrated DMEM medium served as positive control (*n* = 9 eggs). Three days later, the number of blood vessels intersecting a circle of radius 4 mm around the disc was manually counted. Only BM-MSCs could increase in ovo neovascularization in a paracrine way, which was not induced by their EVs. Scale bars represent 4 mm, and data are expressed as mean ± S.E.M. * *p* < 0.05, ** *p* < 0.01, as determined by Kruskal-Wallis test with Dunn’s post-hoc test.

Finally, the paracrine potential of DPSCs and BM-MSCs to stimulate the in vivo formation of new functional blood vessels was tested in the CAM assay (Figure 5). Therefore, the CAM was incubated with Matrigel droplets containing 25X CM or EVs derived from both cell types during three days. Afterwards, eggs were photographed and the number of blood vessels growing towards these droplets was counted. 25X concentrated CM of BM-MSCs was able to enhance in ovo neovascularization (26 ± 2 vs. 21 ± 1 blood vessels for BM-MSC CM compared to negative control). Soluble secreted factors played a major role in this angiogenic effect, since BM-MSC-derived EVs alone had no significant impact on the number of blood vessels. In contrast, CM of DPSCs and their associated EVs could not induce in vivo angiogenesis at the concentrations used in this experimental set-up.

## 4. Discussion

The field of oral and maxillofacial medicine urgently requires new therapeutic strategies to functionally and structurally regenerate damaged dental tissues. Stem cells are a powerful tool in this respect because of their self-renewal and ability to differentiate into other cell types. As a consequence, the potential use of MSC transplantation for tissue regeneration has already been closely studied. Besides their wide differentiation capacity, MSCs could also be beneficial due to their paracrine effects on angiogenesis. MSCs from various tissue sources, including bone marrow, umbilical cord and dental pulp, not only secrete proangiogenic factors, but can also induce in vitro and in vivo neovascularization [8,9,14]. However, because of the drawbacks associated with the use of stem cells themselves as a therapy, focus has recently been shifted to their EVs. Several studies on MSC-derived EVs, especially from the bone marrow, have shown promising functional improvements in animal models for myocardial infarction [19,20], neurological disorders, such as traumatic brain injury [33] and stroke [34], hind limb ischemia [21] and wound healing [35], induced by their proangiogenic properties. In contrast, tumour growth could be inhibited by reducing neovascularization [36,37]. Although, DPSCs have superior proliferation and immunomodulation properties, compared to BM-MSCs [12,13], studies on the angiogenic capacity of their EVs are scarce. Therefore, the aim of this study was to compare in depth the biology and functional angiogenic effects on the level of endothelial cell chemotaxis, and in ovo neovascularization between DPSCs and BM-MSCs and their associated EVs. 

DPSC- and BM-MSC-derived EV bulk pellets, containing small to large particles and possibly co-precipitated proteins, were isolated based on differential ultracentrifugation and filtration. This isolation technique enabled us to identify whether proteins and angiogenic functions were mainly associated with EV-enriched or EV-depleted fractions, without discriminating EV particles, based on origin and size. Moreover, the high particle yield associated with this isolation protocol permits the detection of potential small biological effects. Although, none of the available EV isolation methods can offer a high recovery together with high specificity [32], our isolation protocol resulted in a heterogeneous EV population of MSC origin, with no detectable cell contaminants, as analysed by HR-FCM, western blot and TEM. HR-FCM showed a difference in relative abundancy of CD73-positive EVs between both cell types. NTA revealed a typical size distribution profile between 50 and 300 nm with a mean diameter of about 150 nm for both EV types. However, the fraction of small particles (30–100 nm) was larger for DPSCs, while BM-MSCs secreted a higher percentage of intermediate particles (100–300 nm). In addition, particle numbers per cell were lower for DPSCs compared to BM-MSCs. Casado et al. could demonstrate a positive correlation between the number and size of cell protrusions and secreted EVs from adipose tissue-derived MSCs by combining live atomic force microscopy and scanning electron microscopy [38]. This interaction could be a possible explanation for the observed size and concentration differences between DPSC and BM-MSC EVs, since BM-MSCs have a larger active cell surface area. 

To be able to exert angiogenic effects, EVs may contain molecules associated with the angiogenic process. Several proteins and pathways have already been identified as responsible mediators for the functional effects of MSC-derived EVs on endothelial cells. For example, human umbilical cord MSC-derived EVs could induce angiogenesis by Wnt4/β-catenin signalling [39], while human BM-MSC EVs stimulated in vitro tube formation via the NF-κB pathway [40]. In addition, VEGF and VEGF receptor expression could be enhanced in endothelial cells by HIF-1α activation [21,41]. By using an antibody array, we confirmed the presence of both pro- and anti-angiogenic proteins in EVs and EV-depleted CM of DPSCs and BM-MSCs. Detected proteins, such as Angpt-1 and TIMP-1, play a role in all steps of the angiogenic process, i.e., ECM degradation, endothelial cell proliferation, migration and tube formation. In general, the angiogenic profile of the EV-depleted CM from DPSCs was similar to the profile of DPSC CM, as previously studied by our research group [14]. 

Here, five of the highly expressed angiogenic factors were quantitatively measured in the secreted fractions of both stem cells by ELISA. Protein expressions in non-lysed EVs were negligible, proving that all tested EV-associated factors were located intravesicularly and not membrane-associated or co-precipitated during ultracentrifugation. In addition, their concentrations were higher in CM and EV-depleted CM, compared to lysed EVs. Similarly, Chen et al. showed less expression of most tested molecules in microvesicles of umbilical cord-derived MSCs, compared to their parental cells. They suggested a random transfer of angiogenic proteins to EVs, except for the selective accumulation of some angiogenic cytokines playing a major role in their proangiogenic potential [42]. Wysoczynski et al. showed a significant packaging of Angpt-1 in cardiac MSC-derived EVs, which could interact with Tie2 on endothelial cells to induce their migration [43]. A higher expression of Angpt-1 was detected in all secretion fractions of DPSCs compared to BM-MSCs. In contrast, IGFBP-1 and -3 were more expressed by BM-MSCs. The role of these IGFBP isoforms in angiogenesis is still controversial, since both pro- and anti-angiogenic effects have been observed in vitro and in vivo [44,45]. TIMP-1 was more secreted in CM of BM-MSCs compared to DPSCs. This protein has an anti-angiogenic function by blocking the ECM degrading action of MMPs [46]. Karaöz et al. showed a higher protein secretion of proangiogenic factor VEGF by DPSCs compared to BM-MSCs [12]. However, no significant differences between both stem cells could be detected in our study. Direct comparison of protein concentrations with other studies is impeded by variations in used stem cell types, cell culture conditions and EV isolation protocols between research groups [12,14,42]. Although, this study only focused on the protein content of DPSC- and BM-MSC-derived EVs, proangiogenic properties have also been assigned to miRNAs transported by EVs. Transfer of miRNA-126 from mouse BM-MSC-derived EVs to endothelial cells has been reported to induce in vitro angiogenesis by activation of the PI3K/Akt/eNOS pathway and upregulation of growth factors, including VEGF and fibroblast growth factor-2 (FGF-2) [47]. In addition, miRNA-125a and miRNA-210 have been described to be at least partially responsible for the in vitro proangiogenic potential of EVs from human adipose-derived MSCs, and mouse BM-MSCs, respectively [48,49]. Our study did not determine whether these previously identified EV-associated angiogenic proteins and miRNAs were also responsible for the observed angiogenic effects of DPSC and BM-MSC EVs, and form the subject of further investigation.

The positive effects of DPSC-derived EVs on endothelial proliferation and tube formation have already been demonstrated [25,26]. In addition, our study proved their chemotactic capacity on endothelial cells, which was more limited compared to BM-MSC EVs. When the results were normalized for the higher particle secretion of BM-MSCs, this difference in migration potential was neutralized. These data suggest that DPSC and BM-MSC particles attract endothelial cells to the same extent, but that BM-MSCs produce more particles. Alternatively, the variation in chemotactic capacity between DPSC and BM-MSC EVs could also be associated with their distinct physical characteristics, which might indicate the presence of different EV subtypes. Although, the subdivision of EVs in exosomes and microvesicles cannot only be made based on size, the DPSC EV fraction might contain more small exosomes, and BM-MSC EVs might primarily be larger microvesicles [50]. Moreover, EV-depleted CM of BM-MSCs had a stronger chemoattractant capacity on endothelial cells at lower concentrations, compared to DPSCs. Therefore, BM-MSCs could be more beneficial than DPSCs with regards to the induction of endothelial cell migration. In contrast, Venugopal et al. showed a better in vitro effect of CM and EVs of DPSCs, compared to BM-MSCs in the field of neuroprotection, which can be attributed to the fact that DPSCs are derived from the neural crest [51]. This positive effect on neuronal survival was confirmed by Jarmalaviciute et al. with exosomes of DPSCs derived from human exfoliated deciduous teeth (SHEDs). Oxidative stress-induced apoptosis of human dopaminergic neurons could be reduced by these exosomes, only when their parental cells were cultured under 3D conditions [52]. 

In accordance with the results on endothelial cell chemotaxis, CM derived from BM-MSCs was able to improve in vivo angiogenesis, while CM of DPSCs had no effect on blood vessel formation in the CAM assay under the tested conditions. Direct application of both stem cells has already been shown to stimulate in ovo neovascularization [14,53,54,55]. In addition, similar results have been observed for human and mouse BM-MSC-derived CM [8,56]. These data suggest that continuous production of angiogenic factors by DPSCs is needed for their in ovo angiogenic potential, which might be caused by the restricted half-life of growth factors present in their CM. The importance of these soluble paracrine mediators is also stressed by the fact that DPSC- and BM-MSC-derived EVs alone were not capable of performing in ovo proangiogenic effects. This lack of angiogenic response was not caused by the species difference of chicken endothelial cells versus human EVs, since Ma et al. could successfully stimulate in ovo neovascularization by EVs derived from human umbilical cord MSCs in the CAM assay. However, this study used EV-depleted serum for EV isolation, while our cells were cultured under serum-free conditions [57]. Differences in the applied scaffolds could also have an impact on the preservation and release of angiogenic factors and the in ovo observed angiogenic effects. In addition, although non-specific inflammatory reactions are usually considered negligible before embryonic day 15 because of the relatively immature immune system of the chicken embryo [58], minor inflammation and an associated secondary angio-proliferative response cannot be excluded. However, both DPSCs and BM-MSCs have anti-inflammatory and immunomodulatory capacities, which might counter this possible inflammatory response of the CAM [59,60].

To test the possible impact of differences in uptake capacity between DPSC- and BM-MSC-derived EVs on their functional effects, their internalization by endothelial cells was measured. EV uptake could be confirmed in a time-dependent way starting from three hours onwards, which is similar to the uptake profile of EVs from human adipose MSCs, as previously described by Xue et al. [61]. Because of the potential variation in EV concentration and labelling efficiency, direct comparison of the total amount of internalized EVs, between both stem cell types, was complicated. Therefore, the fluorescent signals were normalized to the total EV uptake at the final time point. DPSC and BM-MSC EVs were taken up at similar rates by endothelial cells during the first 24 h, suggesting that they influence endothelial cell behaviour in a similar way. 

Previous studies have argued that the paracrine proangiogenic effects of MSCs are predominantly mediated by their EV fraction. Komaki et al. and Nakamura et al. showed an equal induction of endothelial tube formation by CM and EVs derived from MSCs of human placental tissue and bone marrow. The associated EV-depleted CM did not affect this angiogenic step [24,62]. Similar results were also observed for human adipose-derived MSCs [63] and rat BM-MSCs [64]. At the level of endothelial migration, a significant effect for all fractions derived from human BM-MSCs could be demonstrated [24]. However, EV depletion of MSC CM from human adipose tissue reduced its chemotactic capacity on endothelial cells [63]. In contrast, our study suggests that the major paracrine angiogenic effect of DPSCs and BM-MSCs on endothelial cell migration and in ovo neovascularization is mediated by secreted growth factors, not associated with EVs, since the chemoattractant effect of EVs on endothelial cells was rather limited and the depletion of EVs had no effect on the chemotactic potential of the CM. Moreover, CM derived from BM-MSCs could enhance in ovo angiogenesis, while their EVs alone could not exert this effect. Despite extended characterization of our EVs, the used isolation protocol cannot exclude the presence of some co-isolated proteins and lipids which might have interfered with the small observed biological effects of the EV-enriched fraction. In addition, part of the biological effect of EV-depleted CM might be induced by the remaining EV fraction, since complete EV depletion of CM cannot be achieved. Other isolation techniques, including ultrafiltration, could enhance this depletion and confirm the major angiogenic role of soluble factors [50].

EVs reflect the actual status of the parental cell, which is very susceptible to minor fluctuations in internal and external factors, including culture medium [65], passage number [66,67], seeding density [66], serum concentrations [68] and oxygen levels [69,70], which could explain inconsistencies between EV studies. Culturing MSCs under hypoxic conditions has indeed been observed to increase their EV production and functional effects on endothelial cell proliferation and tube formation [69,70]. Similar results have been obtained by the addition of growth factors, such as platelet-derived growth factor (PDGF) to the culture medium or by genetically overexpressing important angiogenic pathways including HIF-1α or Akt signalling in the stem cells [26,57,71]. A recent study of Haraszti et al. could also show a significant impact of serum-free culturing on the number, proteome and activity of BM-MSC-derived EVs, with opposite effects on exosomal and microvesicle fractions [68]. Therefore, more standardisation is needed in EV isolation protocols and culture conditions, in order to be able to compare different findings between studies. Moreover, a profound description of the used experimental set-up is required to pinpoint causes of discrepancy, since some researchers omit important experimental details. For example, our ultracentrifugation process differed from the protocol described by Nakamura et al., which could explain the observed inconsistencies in angiogenic effects. Nevertheless, clear information on the volume in which EVs were solved is missing in their study [24]. In addition, Bian et al. isolated EVs of human BM-MSCs under hypoxic conditions and the angiogenic properties were not compared between EVs, EV-depleted CM and complete CM [19].

Furthermore, isolation and characterization of EVs are expensive and time-consuming, which impairs their use in clinical practice. Instead of culturing cells in two-dimensional (2D) monolayers as performed in our study, different companies have been developing bioreactors to allow large three-dimensional (3D) cell cultures, resembling the in vivo situation. These innovations might help to improve EV particle yield, purity and associated functional potential and increase their attractiveness as therapeutic agents [72,73,74]. We demonstrated that EV-depleted CM had a stronger chemotactic capacity on endothelial cells than the EV-enriched fraction in case cells were cultured in 2D monolayers under normoxic conditions. However, it might be possible that the use of other cell culture conditions (e.g., priming cells with growth factors or hypoxia) or other systems (e.g., microcarriers or hollow fibre bioreactors) could further increase the yield and angiogenic potential of EVs. 

Finally, stem cell-derived EV therapies are limited by their paracrine stimulation of angiogenesis and associated tissue regeneration. In contrast, although whole stem cell therapies could have more adverse side effects, they might have a stronger impact on neovascularization. Several researchers demonstrated that MSCs also differentiate into perivascular cells, in order to stimulate vessel maturation, which cannot be achieved by their associated paracrine EVs [75]. This differentiation potential should also be compared between DPSCs and BM-MSCs and taken into account when choosing the cell type and fraction for regenerative therapies.

## 5. Conclusions

In the present study, we have demonstrated positive paracrine effects for both DPSCs and BM-MSCs on endothelial cell migration and in ovo blood vessel formation, with a stronger potential and EV production for BM-MSCs. While, DPSC- and BM-MSC-derived EVs contain a plethora of angiogenesis-related proteins and exert a chemotactic effect on endothelial cells, they only partially contribute to the paracrine potential of their parental stem cells. Future experiments are needed to identify the EV fraction(s) and associated proteins that are responsible for the observed functional effects of EVs. Moreover, a comparative screening for angiogenesis-related miRNAs should be performed, in order to further explain the observed differences between both EV types. Nevertheless, our study urges the need for additional research before whole stem cell therapy could be replaced by EV-based transplantations in regenerative medicine. 

## Supplementary Materials

The following are available online at https://www.mdpi.com/2073-4409/9/2/312/s1, Figure S1: Quantification of the number of living cells at the time of conditioned medium collection, Figure S2: Quantification of density distribution of EVs from DPSCs and BM-MSCs isolated by differential ultracentrifugation, Figure S3: Characterization of EVs isolated from DPSCs and BM-MSCs by differential ultracentrifugation and their corresponding parental cells using (high-resolution) flow cytometry.
