# Supplementary material for: Angiogenic Effects of Human Dental Pulp and Bone Marrow-Derived Mesenchymal Stromal Cells and their Extracellular Vesicles
Source: Cells. 2020 Jan 28;9(2):312. doi: 10.3390/cells9020312 (PMC7072370; doi:10.3390/cells9020312)
Supplement: Supplementary file 1 [file cells-09-00312-s001.pdf]

Supplemental Figures

Figure S1

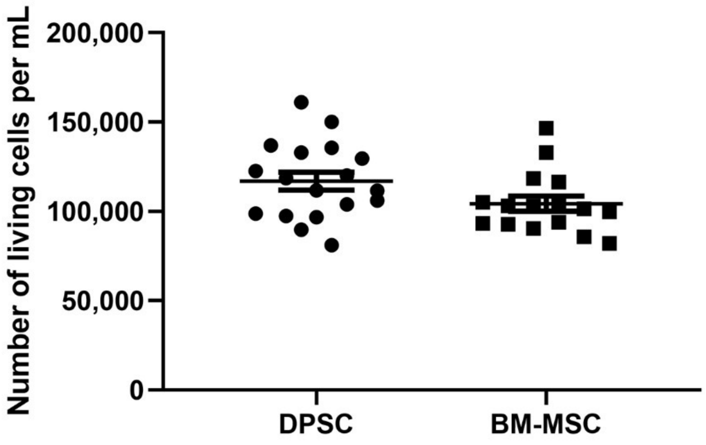

Figure S1: Quantification of the number of living cells at the time of conditioned medium collection. For EV isolation, DPSCs and BM-MSCs were seeded at a density of 100,000 cells per mL. After overnight adherence, cells were placed in serum-free medium for 48 hours. Thereafter, cells were counted using the trypan blue exclusion method. No significant difference in cell number could be detected between both stem cells as analysed by the Mann-Whitney U test. Data are expressed as mean ± S.E.M. (n=18, 9 different donors (DPSCs) and n=16, 3 different donors (BM-MSCs))

Figure S2

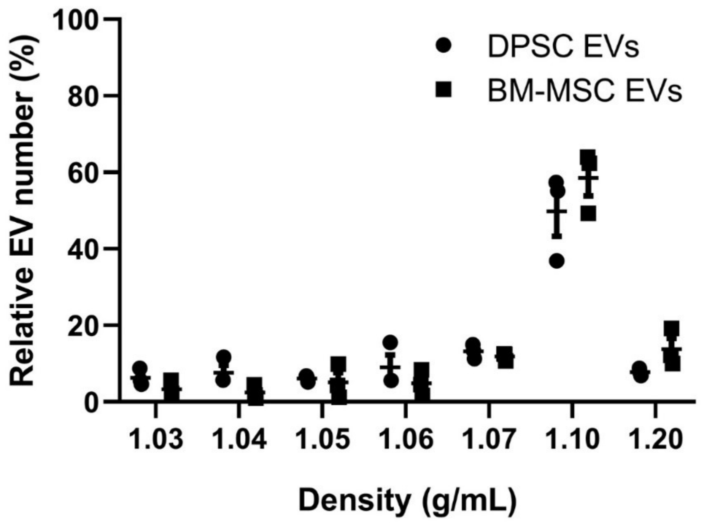

Figure S2: Quantification of density distribution of EVs from DPSCs and BM-MSCs isolated by differential ultracentrifugation. EVs from both cell types were stained with CFDA-SE, and floated into an iodixanol-gradient. EV-containing fractions were diluted in PBS and analysed using high-resolution flow cytometry. Quantitative high-resolution flow cytometric analyses were performed by counting CFDA-positive EVs during a 30-second measurement in the different iodixanol-gradient fractions. The results are presented as the relative number of recorded events to allow for comparison between different donors and cell types (mean ± S.E.M., n=3, 3 different donors). No significant difference in the distribution among the different density fractions between EVs from both cell types was found as determined by two-way ANOVA.

Figure S3

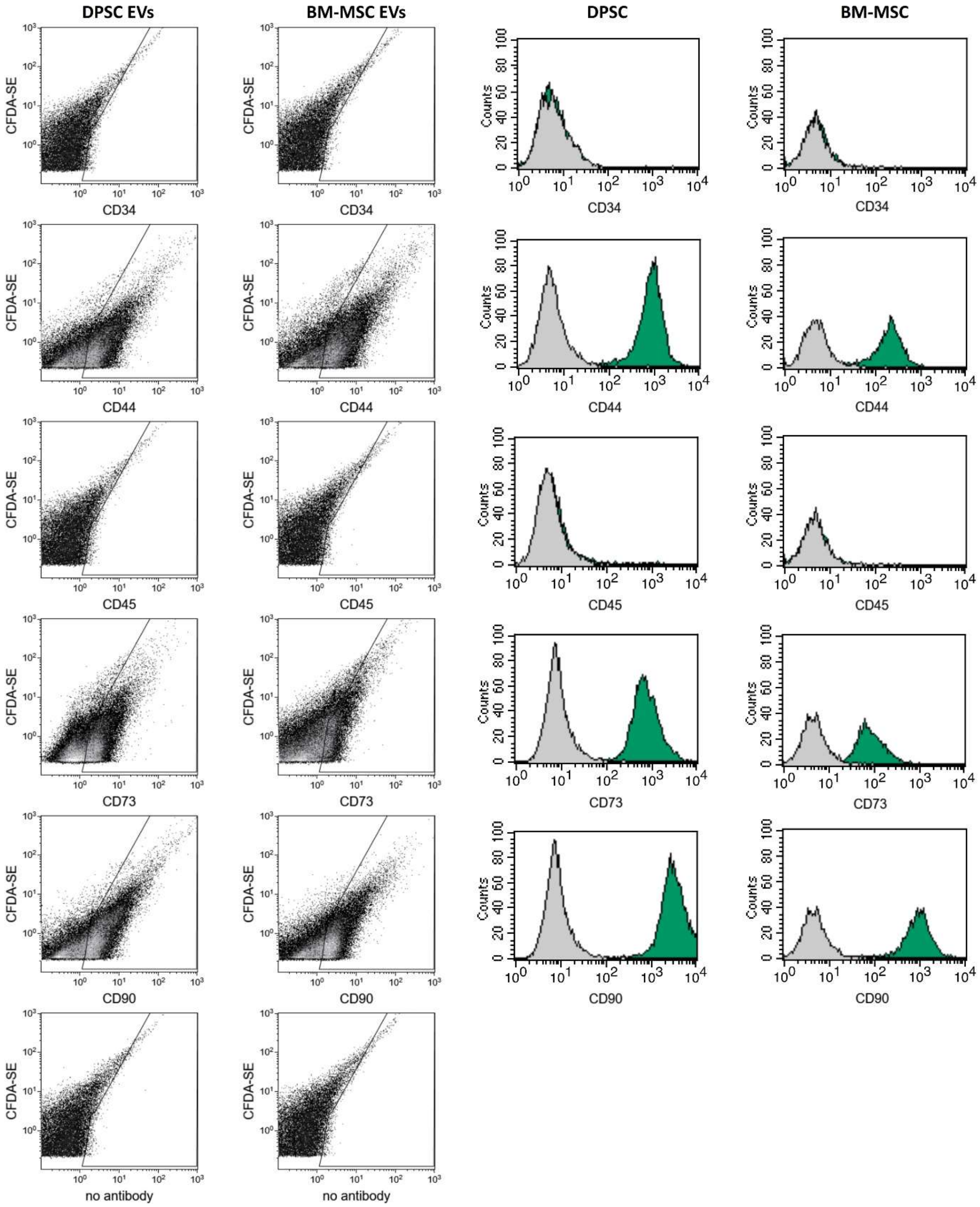

Figure S3: Characterization of EVs isolated from DPSCs and BM-MSCs by differential ultracentrifugation and their corresponding parental cells using (high-resolution) flow cytometry. DPSCs, BM-MSCs and their isolated EVs corresponding with a density of 1.10 g/mL were analysed for their expression of mesenchymal, endothelial and hematopoietic surface antigens CD44, CD73, CD90, CD34 and CD45. Representative dot plots of EVs from both stem cell types (left panels) illustrate the abundance of the markers versus the signal intensity of CFDA-SE labelled EVs (summarizing data are presented in Figure 1B). Representative histograms (right panels) of each marker are also displayed for their parental stem cells (green) with the corresponding isotype controls (grey) as the number of cell counts in function of the fluorescent intensity signal.
